# Whole transcriptome analysis and preliminary construction of ceRNA networks in obstetric antiphospholipid syndrome

**DOI:** 10.3389/fimmu.2025.1663849

**Published:** 2025-12-08

**Authors:** Lan Zhang, Tingting Dong, Xiaoyu Ji, Pengzheng Chen, Xietong Wang

**Affiliations:** 1 Department of Obstetrics and Gynaecology, Shandong Provincial Hospital, Shandong University, Jinan, Shandong, China; 2 Department of Obstetrics and Gynaecology, Shandong Provincial Hospital Affiliated to Shandong First Medical University, Jinan, Shandong, China

**Keywords:** OAPS, exosoma, ceRNA, endothelial dysfunction, inflammation, autophagy

## Abstract

**Background:**

Early diagnosis and therapeutic intervention in obstetric antiphospholipid syndrome (OAPS) are crucial for reducing adverse pregnancy outcomes and improving maternal-fetal safety. This study aimed to investigate the expression profiles of coding and non-coding RNAs in OAPS, as well as the competitive endogenous RNA (ceRNA) network involved in the pathogenesis of OAPS, thereby enhancing our comprehension of the underlying mechanisms of OAPS.

**Methods:**

Plasma samples were collected from 3 OAPS patients and 3 healthy controls. Exosomes were then isolated through differential ultracentrifugation. Comprehensive transcriptome profiling of the purified exosomes was conducted using the Illumina sequencing platform. Differential expression analysis of exosomal messenger RNAs (mRNAs), long non-coding RNAs (lncRNAs), circular RNAs (circRNAs), and microRNAs (miRNAs) was conducted using thresholds set at |log2(fold change) | ≥ 1 and *P* < 0.05. Subsequent bioinformatics analyses included Gene Ontology (GO) and Kyoto Encyclopedia of Genes and Genomes (KEGG) pathway enrichment, and establishment of an lncRNA/circRNA-miRNA-mRNA ceRNA regulatory network.

**Results:**

Exosomes were successfully isolated from both OAPS patients and healthy controls, with subsequent RNA sequencing revealing significant differences in exosomal RNA profiles. Comparative analysis identified 43 differentially expressed mRNAs (DE-mRNAs), 55 DE-lncRNAs, 19 DE-miRNAs, and 72 DE-circRNAs in OAPS-derived exosomes. Integration of these findings enabled the construction of a comprehensive ceRNA regulatory network comprising 15 miRNAs, 14 lncRNAs, 15 mRNAs, and 68 circRNAs. Functional enrichment analysis demonstrated significant associations between these differentially expressed RNAs and critical biological processes, including the AMPK, ErbB, and mTOR signaling pathways.

**Conclusion:**

This study is the first to characterize the distinct exosomal RNA expression profiles in OAPS and construct a ceRNA network related to its pathogenesis. These findings offer novel insights into the molecular mechanisms underlying OAPS and may facilitate the identification of potential biomarkers and therapeutic targets.

## 1 Introduction

Obstetric antiphospholipid syndrome (OAPS) is a thrombophilic autoimmune disorder characterized by persistent antiphospholipid antibodies (aPLs), including lupus anticoagulant (LA), anticardiolipin antibody (aCL), and anti-β2-glycoprotein I antibody (anti-β2GPI). These autoantibodies contribute to adverse pregnancy outcomes such as recurrent early miscarriage, fetal loss, severe preeclampsia, and placental insufficiency (1). The pathogenesis of OAPS involves two main mechanisms: ​​thrombosis​​ and ​​placental dysfunction​​. aPLs interact with endothelial cells, monocytes, and trophoblasts, triggering the release of pro-inflammatory cytokines, activation of the complement system, and overexpression of tissue factor, which collectively impair spiral artery remodeling and placental vascular development (2, 3).Anti-β2GPI antibodies impair trophoblast invasion and syncytialization by suppressing vascular endothelial growth factor (VEGF) and upregulating anti-angiogenic factors like soluble fms-like tyrosine kinase-1 (sFLT-1), leading to placental hypoxia and infarction (4, 5).

Diagnostically, the 2006 Sydney criteria remain the gold standard, requiring at least one clinical criterion (such as ≥3 unexplained early miscarriages or ≥1 fetal death ≥10 weeks) and persistent positivity for aPLs on two occasions ≥12 weeks apart (6). However, clinicians often face uncertainties when treating patients with OAPS or other autoimmune disorders involving specific types of antibodies (7). One major difficulty is that 10–15% of patients with clinical features suggestive of OAPS present with a “seronegative” profile, meaning they test negative for conventional aPLs despite typical symptoms (8). This highlights the limitations of current diagnostic approaches and underscores the urgent need to identify novel biomarkers with potential for broad clinical application. A recent lipidomic study demonstrated that the serum lipid profiles of APS females differ significantly from healthy controls, identifying 12 differentially expressed lipids—particularly triacylglycerols and phosphatidylcholines (PCs). Among these, PC(17:0/22:6) and acylcarnitine (ACar17:3) were proposed as potential serum biomarkers for APS, reflecting altered membrane lipid remodeling and mitochondrial fatty acid metabolism (9).

Exosomes, nanoscale extracellular vesicles (30–150 nm in diameter) derived from various cellular origins through endosomal sorting complex required for transport (ESCRT)-dependent and independent pathways. They mediate intercellular communication by transferring bioactive molecules such as proteins, nucleic acids, and lipids (10, 11).

In pregnancy, exosomes play essential roles in maternal-fetal crosstalk, regulating angiogenesis, apoptosis, immune tolerance, and tissue remodeling—processes fundamental for successful gestation (12). Dysregulation of exosome signaling has been increasingly implicated in pregnancy-related disorders, including preeclampsia and OAPS. Recent immunological and proteomic studies have indicated that extracellular vesicles (EVs), including exosomes, may play a central role in APS pathogenesis by promoting coagulation, endothelial activation, and immune dysregulation (13). Elevated levels of platelet- and endothelial-derived EVs have been detected in APS patients, suggesting they may serve as both biomarkers and mediators of vascular injury (14). Furthermore, proteomic analysis of exosomes from APS patients has revealed enrichment of β2-glycoprotein I (APOH), complement components, and coagulation-related proteins, which may contribute to thrombosis and placental injury (14). Beyond their diagnostic value, exosomes also hold therapeutic potential in pregnancy-related autoimmune conditions. Recent advances have focused on exosome-based therapies, particularly those derived from human umbilical cord mesenchymal stem cells (hucMSC-exos). These exosomes have been shown to alleviate placental injury by delivering miR-146a-5p, which suppresses the TRAF6/NF-κB signaling pathway, thereby reducing the expression of pro-inflammatory cytokines such as IL-1β and IL-18, and modulating apoptosis through the BCL2/BAX pathway (11). Preclinical studies suggest that hucMSC-exos enhance trophoblast migration and promote vascular remodeling in OAPS models, indicating a promising new therapeutic strategy (13). Future research is expected to move toward precision medicine, combining exosomal biomarkers with genetic profiling (e.g., HLA-DR4) to assess patient risk and guide individualized anticoagulation treatment (15). These developments highlight the complex pathophysiology of OAPS and the potential to translate molecular findings into clinical improvements for maternal and fetal health.

In addition to proteins, exosomes contain a variety of substances, such as long non-coding RNAs (lncRNAs), microRNAs (miRNAs), and circular RNAs (circRNAs). This diverse molecular composition enables exosomes to serve as important mediators of intercellular communication by facilitating the horizontal transfer of functional molecules. As a result, exosomes play a key role in maintaining physiological homeostasis and are involved in the development of various pathological conditions (10, 16). LncRNAs, which are linear RNA molecules longer than 200 nucleotides, exert regulatory functions through mechanisms such as chromatin remodeling, transcriptional interference, and modulation of mRNA stability or degradation at the post-transcriptional level (17). Similarly, circ RNAs form covalently closed continuous loops that confer resistance to RNA exonucleases, enabling persistent regulatory effects in target cells (18). LncRNA, circRNA, and mRNA are the main small-molecule substances in the exosome and play essential roles in mediating the biological functions of these vesicles (19).

Therefore, a comprehensive investigation of both non-coding and coding RNAs in plasma-derived exosomes is essential for understanding the molecular mechanisms of OAPS. In this study, we generated RNA-seq datasets for lncRNAs, circRNAs, miRNAs, and mRNAs from plasma exosomes isolated from three OAPS patients and three healthy controls. These data provide a valuable resource for researchers to explore the roles of non-coding and coding RNAs in OAPS and to identify potential imbalances in exosomal RNA profiles under pathological versus normal conditions.

## 2 Methods

### 2.1 Subjects and sample collection

**Figure 1:**
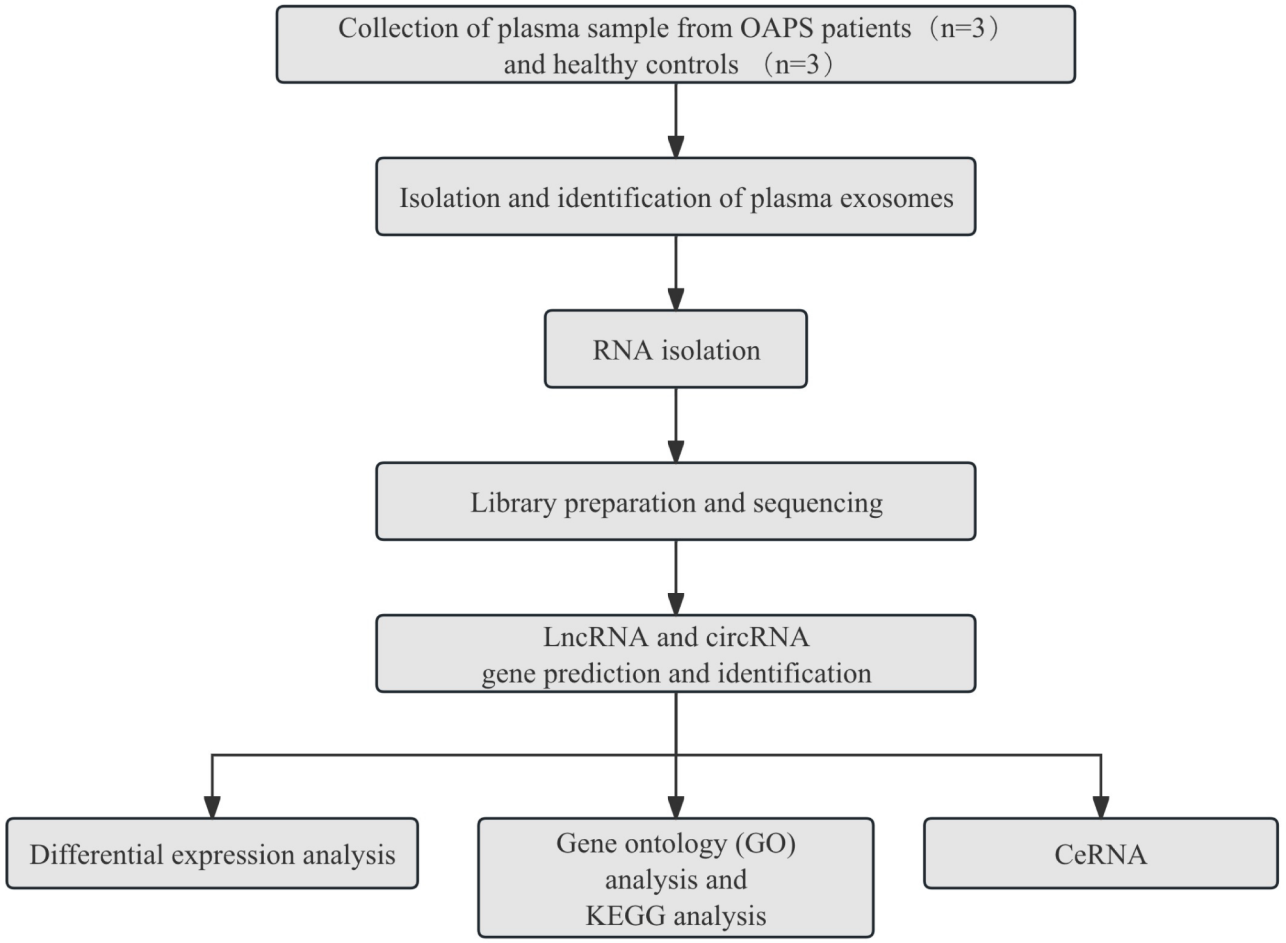
The experimental design and all workflow of this study.

The experimental design and overall workflow as shown in **Figure 1**. To ensure the comparability of results, we established detailed inclusion and exclusion criteria for both the patient and control groups.

### 2.2 Participants and sample collection

This study finally included 3 patients with OAPS (OAPS group) and 3 healthy controls (normal control, NC group). All participants received care at Shandong Provincial Hospital. Peripheral whole blood samples (8 mL) were collected from all subjects in the morning after overnight fasting using EDTA anticoagulant-coated tubes. The blood sample was quickly transferred to the anticoagulant containing heparin and mixed by vortexing or inversion 5 to 10 times.

### 2.3 OAPS group selection criteria

The study cohort comprised patients meeting the following inclusion criteria (20, 21): (1) diagnosis of antiphospholipid syndrome according to international consensus criteria (Sydney classification), confirmed by persistent positivity (≥12 weeks apart) of lupus anticoagulant, anti-cardiolipin antibodies (IgG/IgM), or anti-β2-glycoprotein I antibodies (IgG/IgM) via standardized laboratory assays; (2) age 20–40 years; (3) at 11–14 weeks of pregnancy, the diagnosis of single-child intrauterine pregnancy was confirmed by transvaginal ultrasound and the number of weeks of pregnancy was checked for the patient. Exclusion criteria comprised: (1) clinically significant uterine anomalies (e.g., fibroids ≥4 cm) or adnexal pathology (e.g., ovarian cysts >5 cm); (2) active systemic infections, endocrine disorders requiring pharmacological management (e.g., uncontrolled diabetes mellitus, thyroid dysfunction), or hematological comorbidities (e.g., inherited thrombophilia, coagulation factor deficiencies); (3) pre-existing autoimmune disorders (e.g., systemic lupus erythematosus, rheumatoid arthritis) or cardiovascular/renal/hepatic dysfunction; (4) multifetal gestation or nonviable pregnancy; (5) non-criteria (atypical) OAPS cases; (6) patients with other pregnancy-related complications or comorbidities(e.g., preeclampsia, gestational hypertension, gestational diabetes).

### 2.4 NC group selection criteria

The NC group inclusion criteria comprised: 1) individuals aged 20–40 years; 2) at 11–14 weeks of pregnancy, the diagnosis of single-child intrauterine pregnancy was confirmed by transvaginal ultrasound, and the number of weeks of pregnancy was checked for the patient; 3) absence of a documented history of spontaneous abortion, pre-eclampsia, eclampsia, or preterm birth. Exclusion criteria involved: 1) concurrent diagnoses of gynecological disorders (uterine fibroids, ovarian cysts), endocrine dysfunction, or active infectious conditions; 2) laboratory-confirmed antiphospholipid antibody positivity through standardized immunoassay testing. 3) any autoimmune, hematologic, cardiovascular, renal, or hepatic disorders; 4) multiple or nonviable pregnancies; 5) any pregnancy-related complications or comorbidities identified during the current gestation. This selection protocol ensured the exclusion of potential confounding medical factors while maintaining gestational age homogeneity across the study cohort.

### 2.5 Extraction of exosomes

Exosomes were isolated from plasma samples using a sequential centrifugation protocol as follows (22): First, samples underwent initial centrifugation at 1,500 × g for 10 min at 4°C to eliminate cellular debris and large particulates. The supernatant was then subjected to a second centrifugation at 10,000 × g for 30 minutes at 4°C to eliminate smaller particles using by Thermo Scientific Sorvall Legend X1R(Thermo Fisher Scientific, USA). The resulting supernatant was transferred to clean centrifuge tubes and filtered through a 0.22-μm pore-size (Millipore, USA) membrane to remove any remaining large vesicles. The filtrate was then ultracentrifuged (Beckman Coulter, Optima L-100XP) at 100,000 × g for 60 minutes at 4°C using a fixed-angle rotor to pellet the exosomes (23, 24). Following careful supernatant removal, the exosome-containing pellet was resuspended in 1× phosphate-buffered saline (PBS) and stored at −80 °C until further analysis. This optimized protocol ensures high-purity exosome isolation through a combination of differential centrifugation and size-based filtration (25).

### 2.6 Exocrine identification

Exosome isolation and validation were performed through three complementary analytical approaches: nanoparticle tracking analysis (NTA), transmission electron microscopy (TEM), and western blot (WB) protein profiling.

### 2.7 Nanoparticle tracking analysis

Cryopreserved exosome samples were thawed in a 25 °C water bath and immediately placed on ice to preserve vesicle integrity. For particle size distribution analysis, the exosome suspensions were diluted in 1× phosphate-buffered saline (PBS) (Thermo Fisher Scientific, USA) at an appropriate concentration and directly analyzed using NTA with the NanoSight NS300 system (Malvern Panalytical). All measurements were performed in triplicate at a controlled temperature of 25 °C, with the camera level adjusted to optimize particle visualization.

### 2.8 Transmission electron microscopy

Exosome morphology was assessed by negative staining TEM. Briefly, 10 μL of purified exosome suspension was placed onto Formvar/carbon-coated copper grids (200 mesh) and allowed to adsorb for 1 minute. Excess liquid was carefully removed with filter paper. Samples were subsequently stained with 10 μL of 2% uranyl acetate for 1 minute and air-dried at room temperature. Imaging was performed using a transmission electron microscope operating at 80 kV (JEM-1400, JEOL Ltd).

### 2.9 Western blot

WB analysis was conducted to verify the presence of exosomal marker proteins CD81 and TSG101, and to confirm the absence of the endoplasmic reticulum marker calnexin, indicating minimal cellular contamination. Protein concentrations were determined using the BCA assay (Thermo Fisher Scientific, USA). Equal amounts of total exosomal protein (20 μg per sample per lane) were loaded onto 12.5% SDS–PAGE gels (Epizyme, Shanghai, China), separated, and transferred onto polyvinylidene difluoride (PVDF) membranes (0.22 μm pore size (Millipore, USA)) using a wet transfer system to ensure consistent protein loading across samples. Membranes were blocked with 5% non-fat milk in TBST for 1 hour at room temperature, followed by overnight incubation at 4°C with the following primary antibodies: rabbit monoclonal anti-CD81 (1:1,000; Abcam, ab109201), anti-TSG101 (1:1,000; Abmart, T55985), and anti-calnexin (1:1,000; Cell Signaling, 2679S). After three washes with TBST, the membranes were incubated with horseradish peroxidase (HRP)-conjugated goat anti-rabbit IgG secondary antibody (1:5,000; Sabbiotech, L3012) for 1 hour at room temperature. Protein bands were detected using the Pierce ECL Western Blotting Substrate (Thermo Fisher Scientific) and imaged with a ChemiDoc XRS+ system (Bio-Rad).

Immunoblotting results showed strong positive bands for exosomal markers CD81 (~24 kDa) and TSG101 (~44 kDa) in the isolated exosome samples, with signal intensities comparable to those in the positive control group (CL). A positive control group was included using exosomes previously isolated and characterized by our laboratory. These exosomes had been confirmed to express typical exosomal markers (TSG101, CD81) and were used to verify the specificity of antibody detection and the accuracy of exosome identification. In contrast, calnexin (~90 kDa), a negative marker not present in exosomes, was undetectable in the exosome preparations. This expression pattern confirms the successful isolation of exosomes with minimal contamination from the endoplasmic reticulum, following established standards for exosome characterization. This multi-modal characterization approach provides comprehensive validation of exosome identity, morphology, and protein composition, following the MISEV2018 guidelines for extracellular vesicle research (26).

### 2.10 RNA extraction and library construction

RNA Extraction and Purification: Total RNA was extracted from the exosome samples using the Norgen Exosome RNA Purification Kit (N-51000) according to the manufacturer’s protocol. The concentration and integrity of the extracted RNA were evaluated using a NanoDrop ND-2000 spectrophotometer (Thermo Fisher Scientific) and an Agilent Bioanalyzer 2100 (Agilent Technologies, Santa Clara, CA, USA), respectively. The total RNA was further purified using the RNeasy Mini Kit (QIAGEN, 74106).

Microarray Hybridization and Data Processing: Total RNA (50 ng) was mixed with Spike-In controls (Agilent 5188-5282) and labeled using the Low Input Quick-Amp Labeling Kit, One-Color (Agilent 5190-2305) following manufacturer protocols. Briefly, RNA underwent reverse transcription to synthesize cDNA, which was then amplified through T7 RNA polymerase-mediated *in vitro* transcription to produce complementary RNA (cRNA) labeled with Cyanine-3-CTP. Labeled cRNA was purified using the RNeasy Mini Kit (QIAGEN 74106).

The purified and labeled cRNA (1.65 μg) was mixed with 10X Blocking Agent and 25X Fragmentation Buffer (Gene Expression Hybridization Kit, Agilent 5188-5242), incubated at 60°C for 30 minutes, and then hybridized to the LC Human ceRNA Array V1.0 (4x180K, Design ID: 085202) at 65°C for 17 hours. After washing, the array was scanned using an Agilent Scanner G5761A (Agilent Technologies, Santa Clara, CA, USA).

Scanned microarray images were analyzed using Feature Extraction software (version 12.0.3.1; Agilent Technologies, Santa Clara, CA, USA) to extract the raw data. The row data were normalized using the quantile normalization method and further processed with GeneSpring software (version 14.8; Agilent Technologies, Santa Clara, CA, USA). Probes that were detected in at least 80% of the samples within each group were retained for subsequent analysis.

### 2.11 Data analysis

Microarray data analysis was performed using R statistical software (version 3.6.3). Differentially expressed genes (DEGs) between the OAPS group and control group exosome samples were identified through a Student’s *t*-test. Genes meeting the threshold of adjusted *p*-value < 0.05 and absolute log2 fold change (|log_2_FC|) ≥ 1 were classified as statistically significant DEGs. Unsupervised hierarchical clustering of the identified DEGs was performed using the hierarchical clustering algorithm in the *stats* package, and the expression patterns were visualized as a heatmap via the *pheatmap* package (version 1.0.12). Furthermore, a volcano plot was generated using the *ggplot2* package (version 3.3.3) to illustrate the distribution of DEGs based on their statistical significance and fold change.

To explore the biological significance of the identified DEGs, Gene Ontology (GO) functional enrichment analysis and Kyoto Encyclopedia of Genes and Genomes (KEGG) pathway enrichment analysis were performed. These analyses were executed using the OmicStudio online platform (https://www.omicstudio.cn/tool), which applies robust statistical methods to annotate gene functions and identify signaling pathways significantly enriched among the DEGs.

Target binding relationships between miRNAs and their corresponding mRNAs, lncRNAs, and circRNAs were predicted using TargetScan (version 5.0) and miRanda (version 3.3a). The prediction thresholds were set to TargetScan score ≥ 50 and miRanda energy < –10. Only the intersection results from both tools were retained for further analysis. Finally, high-confidence interaction pairs with a TargetScan score ≥ 95 were selected to construct competing endogenous RNA (ceRNA) relationship pairs based on shared miRNA targets. The resulting ceRNA network was visualized using Cytoscape software.

## 3 Results

### 3.1 Participants and sample collection

A total of three patients with OAPS (OAPS group) and three healthy controls (NC group) were enrolled in this study, all of whom received care at Shandong Provincial Hospital (**Table 1**). Written informed consent was obtained from all patients. This study was carried out according to the World Medical Association Declaration of Helsinki and was approved by the ethics committee of Shandong Provincial Hospital affiliated to Shandong University.

**Table 1:** The clinical and demographic data of the pregnant women.

Category	Variable	OAPS1	OAPS2	OAPS3	NC-1	NC-2	NC-3
Demographic data	- Maternal age (years)	27	32	30	27	28	30
	- Gravidity and parity	G3P0A2L0	G7P2A4L2	G4P1A2L1	G3P2A0L2	G2P1A0L1	G1P0
	- Gestational age at sampling (weeks)	11 + 5	12 + 3	12 + 2	11 + 6	12 + 5	12 + 2
	- Mode of conception (natural/assisted reproduction)	Natural	Natural	Natural	Natural	Natural	Natural
	-Number of fetuses	Single	Single	Single	Single	Single	Single
Obstetric outcomes	- Gestational age at delivery (weeks)	38 + 2	37 + 2	36 + 3	38 + 2	39 + 1	39 + 4
	- Live birth/fetal loss	Live birth	Live birth	Live birth	Live birth	Live birth	Live birth
	- Birth weight (g)	3140	3060	3040	3190	3355	3565
Disease-related variable	- Type of aPL positivity (aCL, anti-β2GPI, LA)	aCL, anti-β2GPI	aCL, anti-β2GPI	aCL, anti-β2GPI	None	None	None
	- Number of positive antibodies (single/double/triple)	double	double	double			
Laboratory parameters	aCL IgG	40.1 CU	221.0 CU	69.4 CU			
	anti-β2GPI IgG	256.1 CU	1109.3 CU	428.4 CU			
	aCL IgG(Re-examine)	17.9 CU	154.5 CU	41.4 CU			
	anti-β2GPI IgG(Re-examine)	80.7 CU	860.0 CU	198.0 CU			
Key Laboratory Parameters	APTT	27.2	331.7	35.1	26.4	27	22.8
	Fib	4.26	3.72	3.83	3.5	2.75	3.98
	PLT	249	131	112	192	210	171
Mixed factors	Presence of autoimmune disease (e.g., SLE)	None	None	None	None	None	None
	Chronic hypertension/diabetes/thyroid disease	None	None	None	None	None	None

Peripheral blood samples were collected from all participants in the morning after overnight fasting. The blood was quickly transferred into tubes containing heparin as an anticoagulant and mixed gently by vortexing or inverting 5 to 10 times to ensure proper mixing.

### 3.2 Characterization of blood plasma-derived exosomes

**Figure 2:**
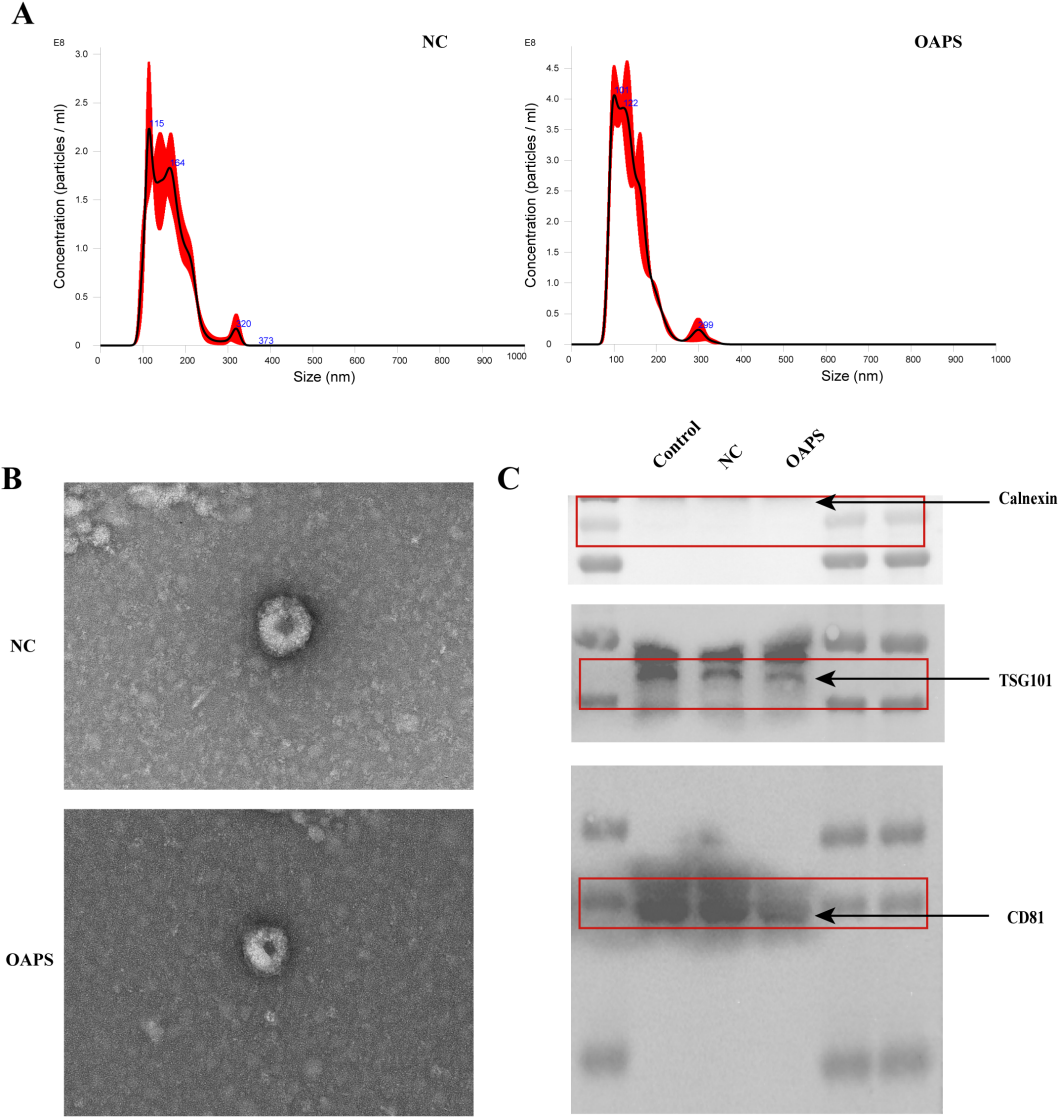
Characterization of exosomes from normal control group (NC) and patients with obstetric antiphospholipid syndrome (OAPS). **(A)** Nanoparticle tracking analysis (NTA) showed the size distribution and concentration of exosomes isolated from NC and OAPS samples. **(B)** Transmission electron microscopy (TEM) images of exosomes from NC and OAPS samples confirmed the typical cup-shaped morphology. **(C)** Western blot analysis of exosome markers, including Calnexin (negative control), TSG101, and CD81. A positive control consisting of exosomes previously isolated and characterized in our laboratory was included to verify antibody specificity and confirm the accuracy of exosome identification. Exosome-related proteins were enriched in both NC and OAPS groups, with no Calnexin contamination observed.

The average peak size of exosomes from control and OAPS blood samples was 159.8 ± 2.8 nm and 140.3 ± 0.4 nm, respectively (**Figure 2A**), consistent with the known size range of exosomes. TEM showed that the extracted substance was nearly round or cup-shaped with an approximate diameter of 100 nm (**Figure 2B**). Western blot analysis confirmed the presence of exosome-specific markers CD81 and TSG101, while the endoplasmic reticulum marker calnexin, used as a negative control, was absent (**Figure 2C**).

Collectively, these findings indicate that exosomes were successfully isolated from the blood samples of both healthy controls and OAPS patients.

### 3.3 Screening and functional enrichment analysis of DE-mRNA

**Figure 3:**
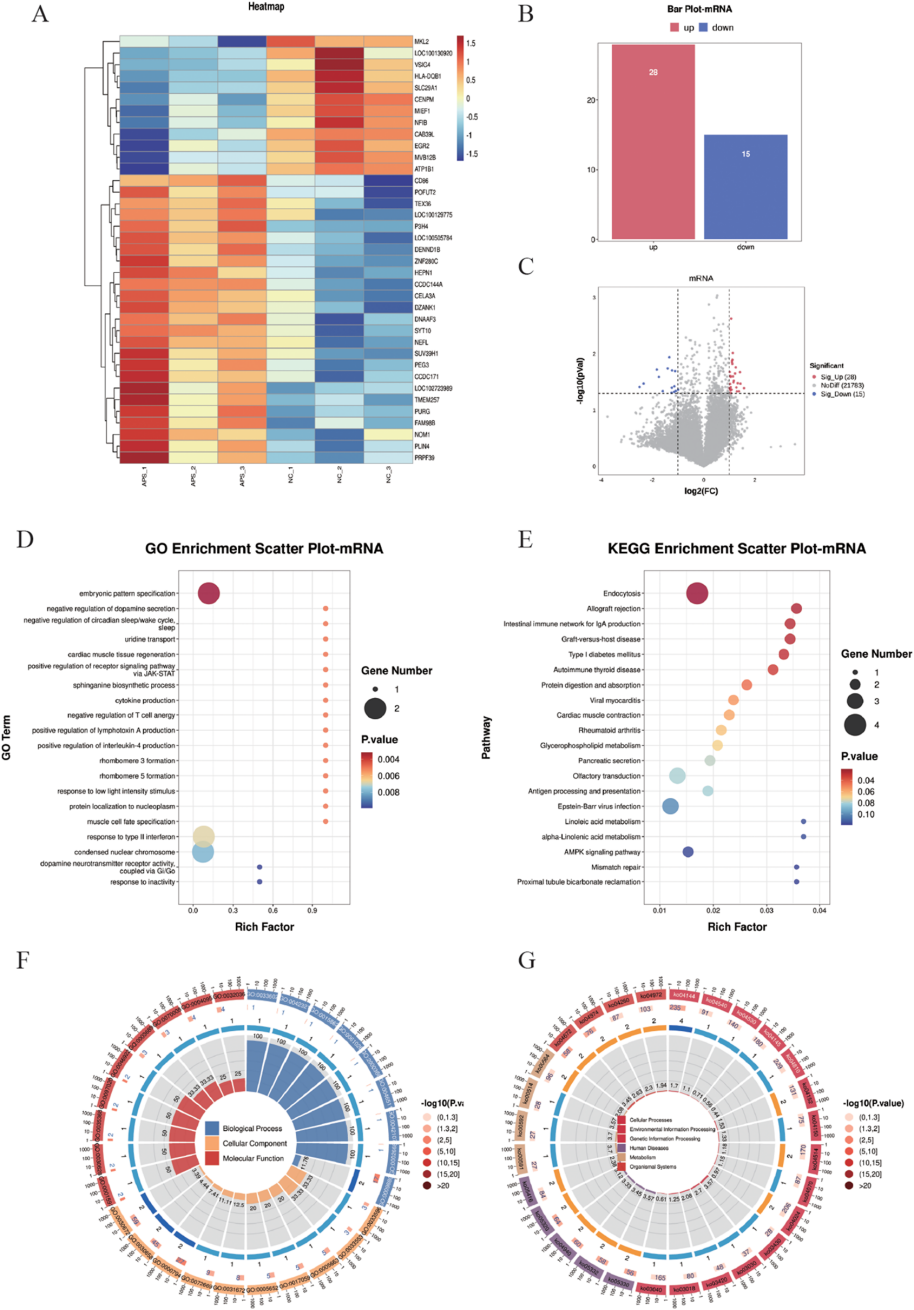
DE-mRNA screening and functional enrichment analysis between OAPS and NC group. **(A)** Heatmap. **(B)** Bar plot of DE-mRNAs statistics. The x-axis represents groups, and the y-axis indicates the number of differentially expressed genes (DEGs) in each group. **(C)** Volcano plot of DE-mRNAs. The x-axis corresponds to log_2_FoldChange, and the y-axis represents −log10(*p*-value). **(D)** GO enrichment analysis of DE-mRNAs. The y-axis displays GO term names, and the x-axis shows the number of enriched genes. **(E)** KEGG enrichment analysis of DE-mRNAs. The y-axis displays KEGG term names, and the x-axis shows the number of enriched genes. **(F, G)**. Loop diagrams illustrating GO enrichment analysis **(F)** and KEGG enrichment analysis **(G)** of DE-mRNAs.

Differentially expressed mRNAs (DE-mRNAs) were subjected to cluster analysis (**Figure 3A**) based on |log_2_FC| ≥ 1 and *P* < 0.05. The results showed that these DE-mRNAs (**Figures 3A–C**) could effectively distinguish between control and OAPS samples. As shown in the bar plot (**Figure 3B**) and volcano plot (**Figure 3C**), a total of 28 genes were significantly upregulated and 15 genes were downregulated in the OAPS group compared to the NC group.

To further investigate the functional implications of the differentially expressed (DE) mRNAs, Gene Ontology (GO) enrichment analysis was performed. The top 20 enriched GO terms were identified, involving 14 genes, including *ERBB4*, *SIM2*, *DRD2*, *SLC29A1*, *SPTLC2*, *TNFSF15*, *CD86*, *EGR2*, *PRPH2*, *NOC2L*, *MYL2*, *SLC30A*8, *P3H4*, and *SUV39H1*. These genes were mainly associated with biological processes such as “embryonic pattern specification,” “positive regulation of receptor signaling pathway via JAK-STAT,” and “cytokine production” (**Figures 3D, F**). In addition, Kyoto Encyclopedia of Genes and Genomes (KEGG) pathway enrichment analysis was conducted, and the top 20 KEGG pathways were summarized. These pathways involved 18 genes, including *ERBB4*, *HSPA1L*, *MVB12B*, *AGAP1*, *HLA_DQB1*, *CD86*, *CELA3A*, *ATP1B1*, *MYL2*, *DGKK*, *PLA2G6*, *OR3A2*, *OR2L2*, *OR8B4*, *CD19*, *CPT1C*, *CAB39L*, and *RFC3*. Notably enriched pathways included “Allograft rejection,” “Protein digestion and absorption,” and the “AMPK signaling pathway” (**Figures 3E, G**).

### 3.4 Screening and functional enrichment analysis of DE-lncRNA

**Figure 4:**
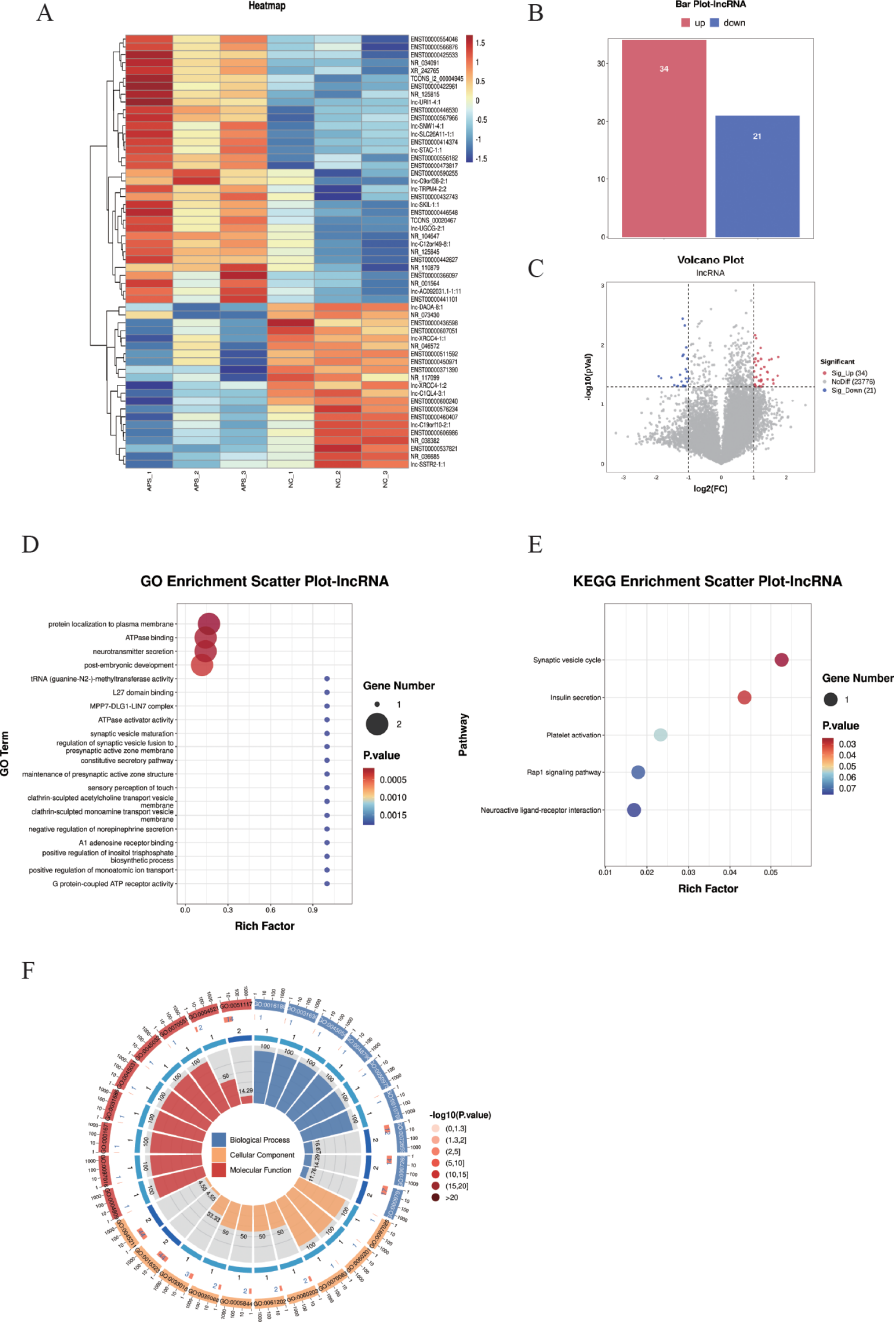
DE-lncRNA screening and functional enrichment analysis between OAPS and NC group. **(A)** Heatmap. **(B)** Bar plot of DE-lncRNAs statistics. The x-axis represents groups, and the y-axis indicates the number of differentially expressed DE-lncRNAs in each group. **(C)** Volcano plot of differentially expressed lncRNAs, the x-axis corresponds to log_2_FoldChange, and the y-axis represents −log10(*p-*value). **(D)** GO enrichment analysis of differentially expressed lncRNAs. **(E)** KEGG pathway enrichment analysis of differentially expressed lncRNAs. **(F)** Loop diagrams showing classification of enriched GO terms under biological process, cellular component, and molecular function categories; outer ring labels GO terms, and color intensity represents enrichment significance -log_10_(*p*-value).

Differentially expressed long non-coding RNAs (DE-lncRNAs) were identified using the criteria of |log_2_FC| ≥ 1 and *P* < 0.05, and subjected to hierarchical clustering analysis (**Figure 4A**). The results demonstrated that these DE-lncRNAs could differentiate between the control and OAPS samples (**Figures 4A–C**). The bar plot (**Figure 4B**) and volcano plot (**Figure 4C**) showed that, compared to the NC group, there were 34 significantly upregulated genes and 21 downregulated genes in OAPS.

The top 20 GO terms were summarized; there were biological processes such as “protein localization to plasma membrane”, “ATPase binding”, “neurotransmitter secretion”, “post-embryonic development”, and “c tRNA (guanine-N2-)-methyltransferase activity” (**Figures 4D, F**).

The KEGG pathways were summarized, and pathways such as “Synaptic vesicle cycle”, “Insulin secretion”, “Platelet activation”, “Rap1 signaling pathway”, and “Neuroactive ligand-receptor interaction” were included (**Figures 4E**).

### 3.5 Screening and functional enrichment analysis of DE-circRNA

**Figure 5:**
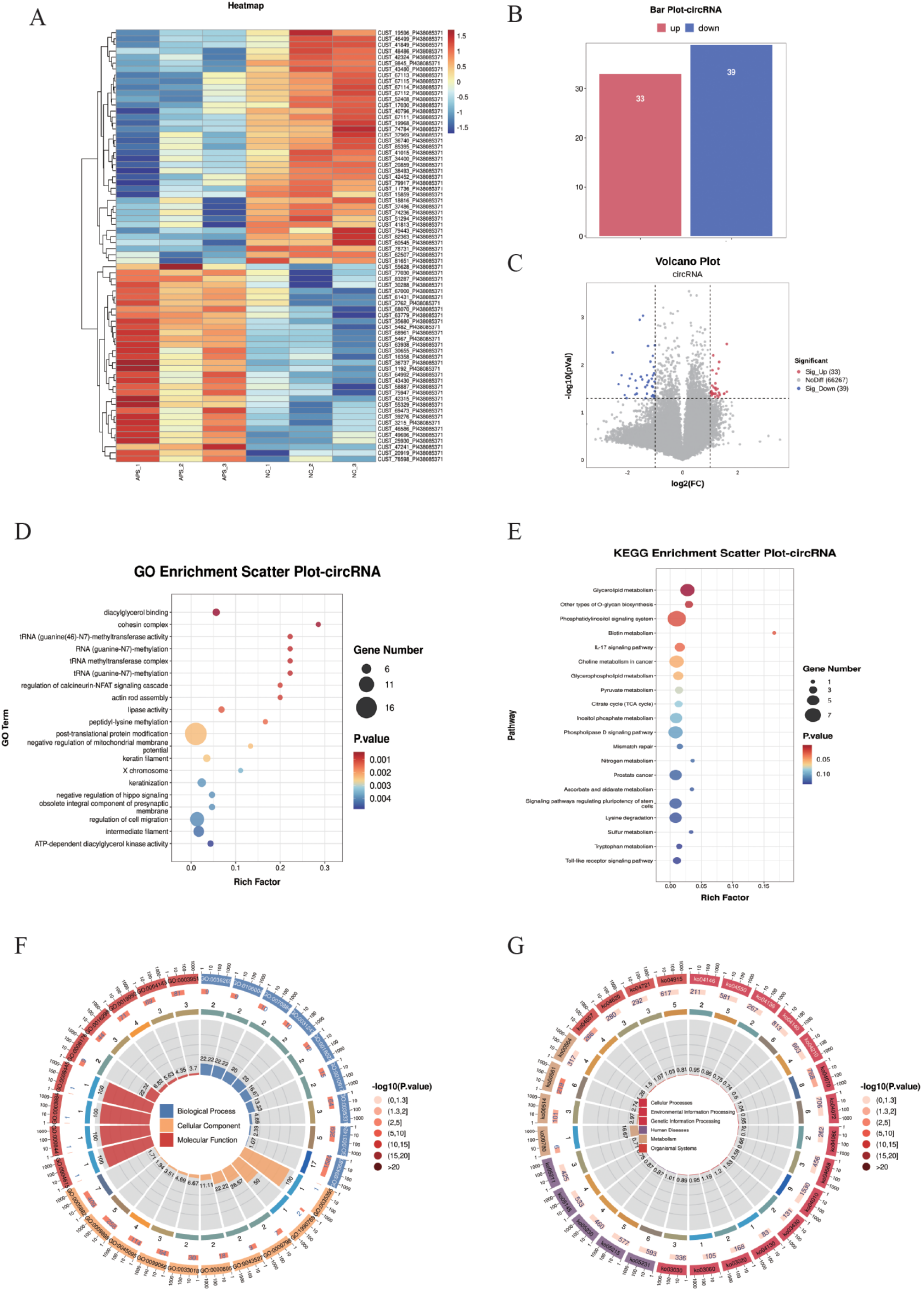
DE-circRNA screening and functional enrichment analysis between OAPS and NC group. **(A)** Heatmap. **(B)** Bar plot of DE-circRNAs statistics. The x-axis represents groups, and the y-axis indicates the number of DE-circRNAs in each group. **(C)** Volcano plot of DE-circRNAs. The x-axis corresponds to log_2_FoldChange, and the y-axis represents −log10(*p*-value). **(D)** GO enrichment analysis of DE-circRNAs. The y-axis displays GO term names, and the x-axis shows the number of enriched genes. **(E)** KEGG enrichment analysis of DE-circRNAs. The y-axis displays KEGG term names, and the x-axis shows the number of enriched genes. **(F, G)** Loop diagrams illustrating GO enrichment analysis **(F)** and KEGG enrichment analysis **(G)** of DE-circRNAs.

Differentially expressed circular RNAs (DE-circRNAs) were subjected to cluster analysis (**Figure 5A**) based on |log_2_FC| ≥ 1 and *P* < 0.05. The results showed that these DE-circRNAs (**Figures 5A–C**) could effectively distinguish between control and OAPS samples. The bar plot (**Figure 5B**) and volcano plot (**Figure 5C**) showed that, compared to the NC group, there were 33 significantly upregulated genes and 39 downregulated genes in OAPS.

Enrichment analysis was performed on the DE-circRNAs. The top 20 GO terms were summarized; among them, there were biological processes such as “diacylglycerol binding”, “RNA (guanine-N7)-methylation”, “regulation of cell migration”, “ATP-dependent diacylglycerol kinase activity”, and “negative regulation of mitochondrial membrane potential” (**Figures 5D, F**).

The top 20 KEGG pathways were summarized. Pathways such as “Glycerolipid metabolism”, “Phosphatidylinositol signaling system”, “Biotin metabolism “, “Phospholipase D signaling pathway”, and “Toll-like receptor signaling pathway” were included (**Figures 5E, G**).

### 3.6 Screening and functional enrichment analysis of DE-miRNA

**Figure 6:**
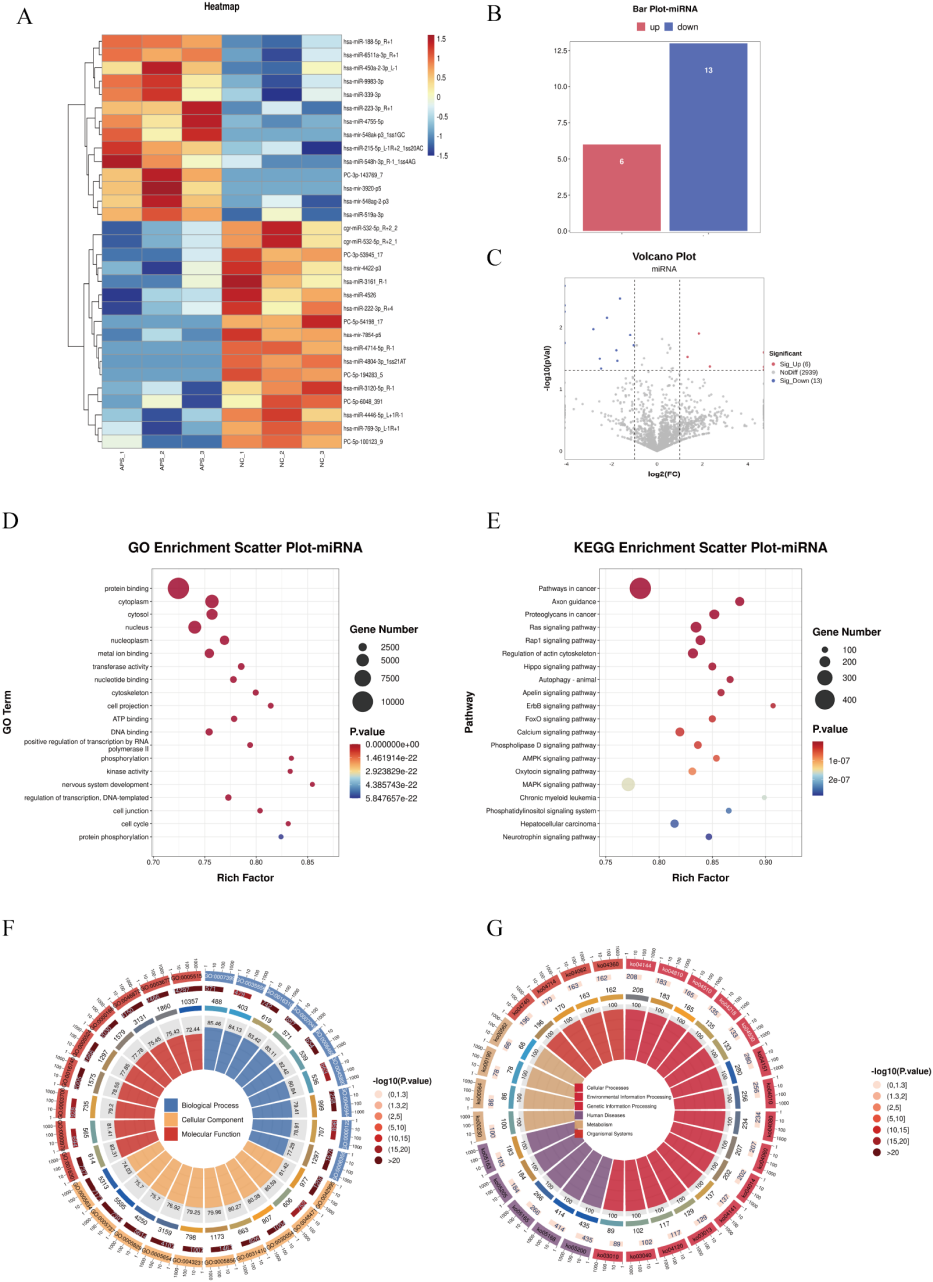
DE-miRNA screening and functional enrichment analysis between OAPS and NC group. **(A)** Heatmap. **(B)** Bar plot of DE-miRNAs statistics. The x-axis represents groups, and the y-axis indicates the number of DE-miRNAs in each group. **(C)** Volcano plot of DE-miRNAs. The x-axis corresponds to log_2_FoldChange, and the y-axis represents −log10(*p*-value). **(D)** GO enrichment analysis of DE-miRNAs. The y-axis displays GO term names, and the x-axis shows the number of enriched genes. **(E)** KEGG enrichment analysis of DE-miRNAs. The y-axis displays KEGG term names, and the x-axis shows the number of enriched genes. **(F, G)** Loop diagrams illustrating GO enrichment analysis **(F)** and KEGG enrichment analysis **(G)** of DE-miRNAs.

Differentially expressed microRNAs (DE-miRNAs) were subjected to cluster analysis (**Figure 6A**) based on |log_2_FC| ≥ 1 and *P* < 0.05. The results showed that these DE-miRNAs (**Figures 6A–C**) could effectively distinguish between control and OAPS samples. The bar plot (**Figure 6B**) and volcano plot (**Figure 6C**) showed that, compared to NC, there were 6 significantly upregulated genes and 13 downregulated genes in OAPS.

For the screened DE miRNAs, biological processes of GO terms and KEGG signaling pathways were analyzed, and with *P* < 0.05 as the enrichment significance threshold, 20 GO terms of biological processes and 20 KEGG pathways were significantly enriched. The enriched GO terms included “protein binding,” “metal ion binding,” “transferase activity,” “nucleotide binding,” and “positive regulation of transcription by RNA polymerase II” (**Figures 6D, F**). The significantly enriched KEGG pathways included the “Ras signaling pathway,” “Regulation of actin cytoskeleton,” “Hippo signaling pathway,” “ErbB signaling pathway,” “Phospholipase D signaling pathway,” and “AMPK signaling pathway” (**Figures 6E, G**).

### 3.7 Characterization and functional analysis of ceRNA regulatory networks

**Figure 7:**
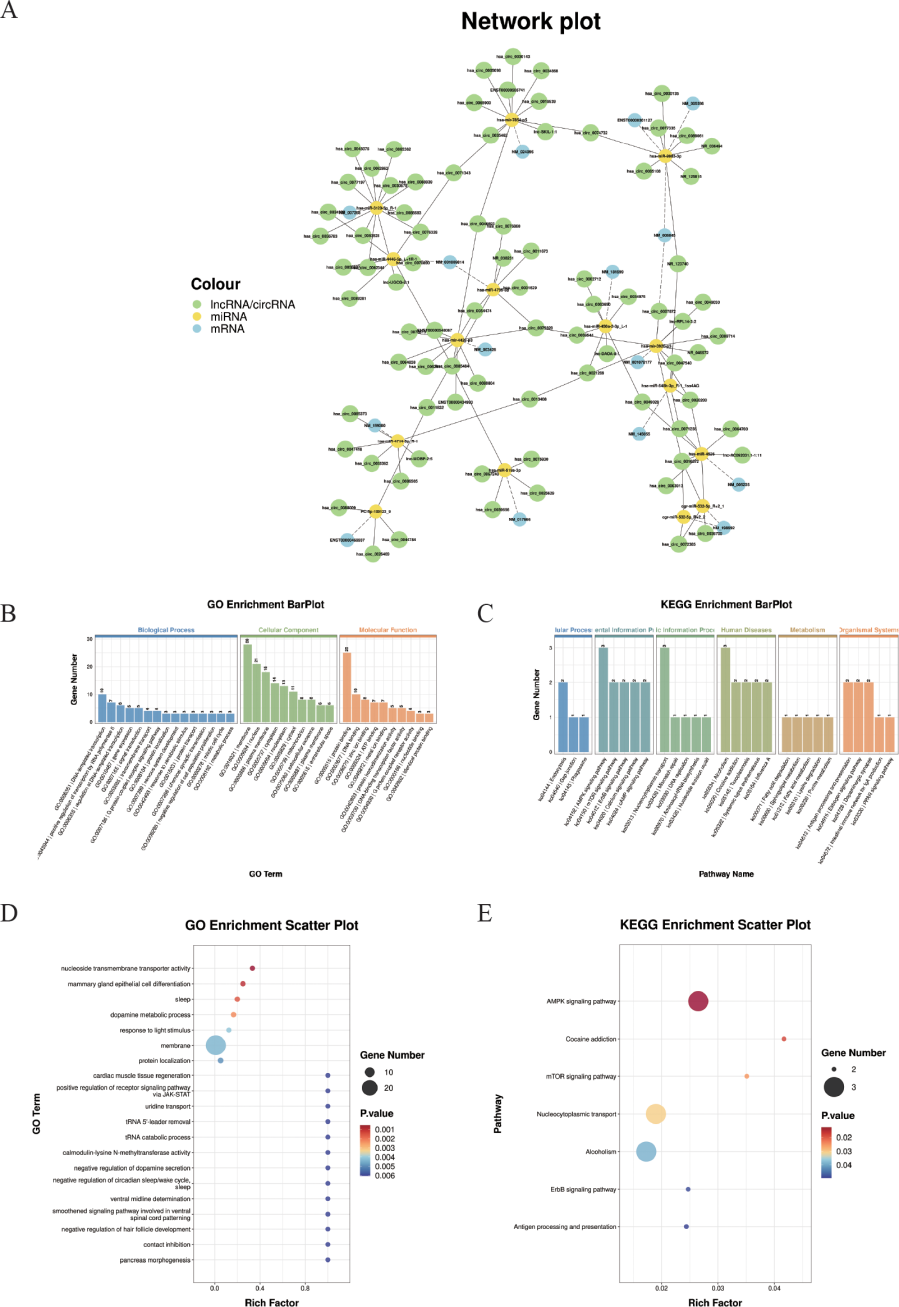
*Construction of the ceRNA regulatory network. **(A)** Illustrates the competing endogenous RNA (ceRNA) network comprising circRNAs, lncRNAs, miRNAs, and mRNAs. Nodes represent transcripts (green: circRNAs and lncRNAs; yellow: miRNAs; blue: mRNAs). This network highlights potential ceRNA regulatory axes involved in the studied pathological process.***(B)** GO enrichment bar plot of ceRNA-related genes. The x-axis displays GO term names (biological processes, cellular components, and molecular functions), and the y-axis shows the number of enriched genes. **(C)** KEGG enrichment bar plot of ceRNA-related genes. The x-axis displays KEGG pathway names, and the y-axis shows the number of enriched genes. **(D)** GO enrichment scatter plot. **(E)** KEGG enrichment scatter plot.

A total of 31 miRNAs, 15 mRNAs,18 lncRNAs, and 101 circRNAs were obtained through screening relationships. TargetScan (5.0) and Miranda (3.3a) software were used to predict the targeting binding relationship between miRNA and mRNA, and lincRNA, respectively, and the intersection of the two software was taken. A ceRNA regulatory network composed of 15 miRNAs, 14 lncRNAs, 15 mRNAs, and 68 circRNAs was constructed based on the common miRNA combination. Cytoscape software was used to visualize the network (**Figure 7A**).

Subsequently, the genes involved in the ceRNA network were subjected to GO term and KEGG pathway enrichment analyses. (**Figures 7B-E**). A total of 20 GO biological processes and 7 KEGG pathways were significantly enriched (*P* < 0.05). As shown in **Figure 7D**, “ negative regulation of dopamine secretion”, “ uridine transport”, “ cardiac muscle tissue regeneration”, “ positive regulation of receptor signaling pathway via JAK-STAT”, and “ pancreas morphogenesis” were significantly enriched.

In addition, the 7 KEGG pathways include the “ AMPK signaling pathway”, “ mTOR signaling pathway”, “ ErbB signaling pathway”, “ Antigen processing and presentation “, “ Cocaine addiction”, “ Nucleocytoplasmic transport “, and “Alcoholism”. (**Figure 7E**).

## 4 Discussion

OAPS is a thrombophilic autoimmune disorder characterized by persistent aPLs, including LA, aCL, and anti-β2GPI, leading to pregnancy complications such as recurrent miscarriage, intrauterine fetal demise, preeclampsia, and fetal growth restriction. Early diagnosis and intervention are critical, motivating efforts to identify reliable biomarkers and elucidate underlying pathophysiological mechanisms.

Emerging evidence highlights the diagnostic potential of exosomal molecular components—miRNAs, lncRNAs, circRNAs, and proteins—as novel biomarkers. In this study, plasma-derived exosomes from OAPS patients and healthy controls were subjected to RNA sequencing. We identified 55 DE-lncRNAs, 72 DE-circRNAs, 43 DE-mRNAs, and 19 DE-miRNAs. A competitive endogenous RNA (ceRNA) network revealed interactions among 15 miRNAs, 14 lncRNAs, 15 mRNAs, and 68 circRNAs. Functional enrichment analysis indicated significant associations with 177 GO terms and seven KEGG pathways, including AMPK signaling, ErbB signaling, antigen processing and presentation, mTOR signaling, and nucleocytoplasmic transport.

OAPS pathogenesis is mainly driven by aPLs, particularly anti-β2GPI-DI antibodies (27), which activate endothelial cells, immune components, and complement cascades, promoting thrombosis and inflammation (28).

In 2014, the involvement of the mammalian target of rapamycin (mTOR) signaling pathway in APS pathogenesis was first reported (29). Accumulating evidence indicates that mTOR plays a central role in endothelial dysfunction, autophagy inhibition, inflammation amplification, and pregnancy failure in APS (30). Anti-β2GPI antibodies form immune complexes with β2GPI, markedly activating mTOR in monocytes, which upregulates tissue factor (TF) and interleukin-8 (IL-8), promoting thrombosis and inflammation (31). mTOR activation involves both classical TLR4 signaling and phosphorylation of p38 and ERK1/2 MAPKs. The mTOR inhibitor rapamycin effectively suppresses this cascade, mitigating procoagulant and pro-inflammatory effects in monocytes (28, 31). Additionally, mTOR regulates endothelial autophagy; anti-phospholipid antibody complexes inhibit autophagy via the PI3K/AKT/mTOR pathway, causing endothelial dysfunction and oxidative stress, which exacerbate vascular damage and inflammation in APS (32). Based on these mechanisms, mTOR inhibitors show therapeutic potential in modulating immune inflammation, improving endothelial function, and preventing thrombosis (33). Clinical and preclinical studies are ongoing to assess mTOR inhibitors, especially in patients resistant to conventional anticoagulants, and future large-scale trials will clarify their safety and efficacy, supporting precision treatment strategies.

AMPK (5’-adenosine monophosphate-activated protein kinase) is an energy sensor that maintains cellular metabolic homeostasis and regulates inflammation and vascular endothelial function. Activation of AMPK exerts potent anti-inflammatory and immunomodulatory effects in various cell types and models of autoimmune disease (34). In umbilical vein endothelial cells (HUVEC), activation of AMPK has been shown to inhibit NF-κB activity induced by palmitate or tumor necrosis factor-α (TNF-α) (35) and suppresses angiotensin II–induced STAT1 activation, thereby reducing iNOS and COX-2 expression and decreasing IL-6 and MCP-1 secretion (36). Meanwhile, under conditions of metabolic stress, AMPK exerts anti-inflammatory effects through multiple mechanisms, including the regulation of macrophage-mediated inflammation and improvement of insulin sensitivity (37). Moreover, AMPK enhances endothelial nitric oxide synthase (eNOS) expression and nitric oxide (NO) production, improving vascular dilation and endothelial function (38, 39). AMPK is also closely linked to autophagy regulation. It promotes autophagy by directly activating ULK1 and indirectly by inhibiting mTORC1-mediated suppression of ULK1 (40, 41). Animal studies have suggested that AMPK agonists, such as AICAR and metformin, can significantly reduce inflammation and endothelial injury (42). Notably, metformin synergizes with mTOR inhibitors (e.g., rapamycin) to enhance autophagy, suggesting cross-regulation between the AMPK and mTOR pathways in APS (43). In summary, AMPK exerts multifaceted protective effects in APS, including anti-inflammatory activity, endothelial protection, and promotion of autophagy.

The ErbB signaling pathway consists of receptor tyrosine kinases, including EGFR (ErbB1), HER2 (ErbB2), HER3 (ErbB3), and HER4 (ErbB4), which play key roles in regulating cell proliferation, differentiation, and inflammatory responses (44). Originally studied in tumor biology, this pathway has recently been implicated in endothelial injury and anti-inflammatory regulation. EGFR regulates endothelial proliferation, permeability, and tube formation, while its abnormal activation contributes to oxidative stress–related endothelial dysfunction in diabetes (45). Activated EGFR triggers multiple downstream cascades, including PI3K/AKT/mTOR, RAS/RAF/MEK/ERK, PLCγ/PKC, and JAK2/STAT3 pathways. Through the PI3K–AKT axis, EGFR or HER2 signaling suppresses IFN-γ responses and the expression of interferon regulatory factors and inflammatory chemokines (44). In cardiomyocytes, inhibition of EGFR phosphorylation reduces TNF-α production via the EGFR/p38/ERK1/2 pathway, thereby mitigating inflammation and limiting endothelial–immune interactions (46, 47). ErbB2/ErbB3 is highly expressed in HUVEC and plays an essential role in endothelial cell proliferation and angiogenic responses (48). ErbB2 has been demonstrated to play a crucial role in myocardial development and is associated with the regulation of myocardial cell proliferation (49). In pathological conditions such as pulmonary arterial hypertension, upregulated ErbB3 promotes endothelial dysfunction, whereas ErbB3 knockout alleviates vascular lesions (50). However, the ErbB signaling pathway also protects endothelial cells by regulating adhesion molecules and suppressing inflammation. For example, ErbB4 can reduce the expression of VCAM-1 and E-selectin via the PI3K/AKT pathway, thereby decreasing neutrophil adhesion (51). The NRG-1/ErbB4 signaling pathway can inhibit the PI3K/Akt and STAT3 pathways and reduce the release of pro-inflammatory factors (52). Furthermore, ErbB4 is highly expressed in M1 macrophages, where its ligand NRG4 induces mitochondrial-mediated apoptosis and decreases TNF-α, IL-1β, and IL-6 expression (53). These findings suggest that the ErbB signaling pathway has potential value in regulating endothelial function, vascular remodeling, and anti-inflammatory protection. However, its specific role in vascular and pregnancy-related pathological processes in APS remains unclear.

AMPK, as an energy-sensing kinase, is activated in the state of energy deprivation. It can phosphorylate TSC2 and Raptor, thereby inhibiting the activity of mTORC1, promoting autophagy, inhibiting protein synthesis, and exerting anti-inflammatory effects (54, 55). When the EGFR/HER2 (ErbB family) receptors are activated, they can activate mTORC1 through the PI3K/AKT pathway, thereby promoting cell growth, protein synthesis, and the production of inflammatory factors. This pathway enables mTORC1 to upregulate HIF-1α and VEGF, enhancing angiogenesis and simultaneously supporting pro-inflammatory responses under inflammatory conditions (56). Activated AMPK can directly phosphorylate ErbB (such as EGFR/HER2), inhibiting their kinase activity. This helps enhance the AMPK’s metabolic-promoting and anti-inflammatory functions. Conversely, ErbB activation inhibits AMPK (or the upstream kinase of LKB1) through AKT, forming a negative feedback regulation (55). The AMPK, ErbB (EGFR/HER2), and mTOR signaling pathways form a highly interactive regulatory network that jointly maintains energy metabolism, autophagy, cell proliferation, and the balance of inflammation. In disease states (such as tumors, metabolic disorders, and vascular inflammation), the imbalance between ErbB-mediated mTOR activation and the protective inhibition of AMPK becomes an important mechanism for pathological progression. Understanding this network helps develop multi-target intervention strategies for APS, such as the combined application of AMPK activators and EGFR/mTOR inhibitors.

The strengths of this study include the integration of exosomal RNA sequencing with ceRNA network analysis, enabling a systematic exploration of the molecular mechanisms underlying OAPS. The identification of 15 key miRNAs with no previously reported roles in OAPS highlights the novelty of our findings. KEGG pathway enrichment revealed mechanistic intersections between pathways, providing additional insight into potential regulatory networks relevant to disease pathogenesis. However, several limitations should be acknowledged. The sample size was relatively small, which may limit the generalizability of the findings. External validation in independent cohorts is lacking, and the clinical applicability of the identified RNAs as disease biomarkers remains to be determined. Future studies with larger sample sizes and functional validation are needed to confirm these results and assess their translational potential.

These findings underscore the critical role of exosomal RNAs in the pathogenesis of APS. The constructed ceRNA network offers a valuable framework for elucidating molecular interactions in OAPS and reflects the complex interplay between immune regulation and signaling pathways typical of autoimmune diseases. A deeper understanding of these interactions may not only enhance insights into the underlying mechanisms of OAPS but also provide a basis for identifying novel therapeutic targets and developing new strategies for clinical intervention.

## Data Availability

The data presented in the study are deposited in the NCBI GEO and BioProject repository, accession numbers GSE312344 and PRJNA1372988, respectively.
